# Exploring Pathways to Gambling: Proposing the Integrated Risk and Protective Factors Model of Gambling Types

**DOI:** 10.1007/s10899-020-09929-2

**Published:** 2020-01-21

**Authors:** Natalie L. Hearn, Jane L. Ireland, Mike Eslea, John E. Fisk

**Affiliations:** 1 School of Psychology, University of Central Lancashire, Preston, UK; 2 Ashworth Research Centre, Mersey Care NHS Foundation Trust, Maghull, UK

**Keywords:** Gambling, Pathways model, Gambling forums, Protective factors

## Abstract

Three linked studies, testing key aspects of the Pathways towards Problem and Pathological Gambling Model (Blaszczynski and Nower in Addiction 87(5):487–499, 2002), are presented. Study one comprised 204 students and 490 gambling forum users. It predicted differences in gambling severity, mental health and substance use across different motives for gambling. Those with a primary social motive for gambling displayed less severe gambling and anxiety than those without, with the primary coping subgroup displaying the most anxiety and depression. Those who gambled primarily to enhance positive affect reported severe gambling. Study two comprised 404 gambling forum users and 265 students. Similar groups to the Pathways Model emerged, with a behaviourally conditioned and an emotionally vulnerable group. Unexpectedly, however, those in the emotionally vulnerable group reported more severe cognitive distortions than the behaviourally conditioned group. The final study, 378 gambling forum users and 201 students, found, as predicted, that three distinct gambling groups emerged; (1) those with lower levels of psychopathology and higher levels of protective factors; (2) those with heightened pre-existing anxiety and depression, and moderate levels of protective factors; and (3) those with heightened impulsivity, psychopathology, offending behaviour and the least protective factors. Three gambling groups are consequently presented (Social Gambler; Affect-Regulation Gambler; Antisocial Gambler) alongside the proposed Integrated Risk and Protective Factors Model of Gambling Types (IRPF-MGT). Directions for future research and implications for practice are outlined.

## Introduction

Gambling has represented a recreational activity for several centuries (Raylu and Oei 2004). The availability has certainly increased, moving beyond physical settings, such as casinos, bookmakers, bingo halls, arcades, pubs and clubs, to internet-based forums (Gambling Commission 2017). The growth of the gambling industry has been paralleled by reported increases in problematic gambling and addiction (Nowak and Aloe 2014). Whilst accepting that many engage in gambling without adverse consequences, focus here is on *problem gambling*. This term is presented here as an all-encompassing expression for a plethora of terms used to describe gambling with potential challenges (Potenza et al. 2002). The current paper does not aim to address terminology and, for ease of expression, will use the term problem gambling wherever possible.

Regarding the prevalence of problem gambling, in general populations there are differences noted. Williams et al. (2012) conducted a meta-analysis of 202 studies between the years of 1975 and 2012. The found, dependant on the jurisdiction sampled and the sampling time frame, prevalence rates of problem gambling varied between 0.5 and 7.6%, with an average rate across all countries of 2.3%. The lowest standardised prevalence rates occurred in Europe. This was supported by Conolly et al. (2017) who documented problem gambling to fall at 1.3% for men, 0.2% for women and 0.7% overall. Students have been reported to gamble at more severe levels than the general population (Blinn-Pike et al. 2007). For example, Nowak and Aloe (2014) incorporated over 13,000 students from 18 studies between 2005 and 2013 in their study and estimated the prevalence of probable pathological [problem] gambling to be 10.2%. Nowak and Aloe (2014) argued that students were more susceptible to developing problem gambling due to a range of interacting factors. These were termed *The Five A’s*; *Age*, with students more susceptible to engaging in risky behaviours due to their young age; *Availability* of gambling opportunities, including online gambling; *Acceptability* of gambling amongst society and in student culture; *Advertising*, which promotes gambling and portrays it as a sport/skilled exercise; and *Access* to monetary funds through student loans and overdraft credit allowances.

Although noting prevalence is important in highlighting the scale of the problem, equally important is understanding why individuals gamble. Research has identified multiple motives (Chantal et al. 1995). This includes gambling to experience excitement (Platz and Millar 2001), as a means of coping and/or ‘escaping’ from daily routine (Loroz 2004). For others, it may facilitate socialising (Lee et al. 2006), lead to raised income (Park et al. 2002) and/or maintain optimal levels of arousal (Blaszczynski and Nower 2002; Gupta and Derevensky 1997; Lloyd et al. 2010; Raylu and Oei 2004; Wood and Griffiths 2007).

Gambling motives form a major part of theoretical models for gambling (e.g. Blaszczynski and Nower 2002; Jacobs 1986). Several models are used to explain problematic gambling, including addiction models (Blaszczynski and Nower 2002; Reith and Dobbie 2012), biological perspectives (APA 2013; Jazaeri and Bin Habil 2012), behavioural applications of understanding (e.g. Kassinove and Schare 2001), social learning applications (e.g. Abrams and Kushner 2004; Coventry and Constable 1999; Meyer et al. 2009), and cognitive explanations (e.g. Rickwood et al. 2010; Cunningham et al. 2014). On their own they are unable to provide an integrated understanding of the multitude of factors key to gambling motivation. This has led to a focus on the *Pathways Model of Problem and Pathological Gambling* (PMPPG), proposed by Blaszczynski and Nower (2002). The Pathways Model is an integrated model that acts as a framework for understanding three different pathways to problematic gambling, each characterised by distinct psychological variables.

The first pathway, *behaviourally conditioned*, argues that problematic gambling develops through environmental factors and behavioural reinforcements, rather than through psychopathology. Individuals in this pathway frequently fluctuate between normal, heavy and excessive gambling due to repeated exposure to gambling activities, cognitive distortions regarding winning prospects and personal skill, and poor decision-making, rather than because of impaired control. They can also report substance misuse issues, anxiety and depression, but these are considered a consequence of gambling and not the cause (Blaszczynski and Nower 2002). Research has further suggested how the prospect of winning, combined with increased arousal and subjective excitement, contributes significantly to this pathway (Ledgerwood and Petry 2010).

The second pathway, *emotionally vulnerable*, has similar antecedents to the first through the availability of gambling. This subsequently includes conditioning, cognitive processes and cognitive schemas. However, this group arguably gamble to reduce pre-existing negative affect (e.g. stress, anxiety and depression). They are considered to have poor emotional coping and problem-solving skills, low self-esteem and to have suffered negative life events (Blaszczynski and Nower 2002). The third pathway, *antisocial*-*impulsivist,* presents with similarities to the emotionally vulnerable pathway. However, they also have an increased likelihood of attention deficit and a history of antisocial personality and behaviours. Individuals in this pathway display increased traits of impulsivity and are more likely to engage in non-gambling related criminal acts and substance misuse. Even though arousal and excitement are linked with this and the prior pathways, it is thought to have more significance with the *antisocial*-*impulsivist* pathway (Blaszczynski and Nower 2002).

The Pathways Model, although benefited by incorporation of several risk factors into an integrated model, is not without criticism. For example, it considers only risk factors and does not attempt to capture protective factors, which could protect against a problematic pathway emerging and/or continuing (Dickson et al. 2002, 2008; Lussier et al. 2007). Research investigating the model is limited (Valleur et al. 2016) and although the pathways have some support, there are also inconsistencies regarding the number and nature of the groups it presents (e.g. Lobo et al. 2014; Nower et al. 2012; Stewart and Zack 2008; Suomi et al. 2014).

Several studies have further identified a group of gamblers that lack any *severe* levels of psychopathology and who gamble due to socialisation and conditioning processes (Stewart and Zack 2008; Suomi et al. 2014). This group appear similar to the behaviourally conditioned group (Blaszczynski and Nower’s 2002), regarding their lack of severe psychopathology, but not all studies have found a similar subgroup (e.g. Ledgerwood and Petry 2006; Lesieur and Blume 1991; Zimmerman et al. 1985). In addition, a second group appearing to emerge is one characterised by increased levels of depression, anxiety, stress and/or other debilitating affective states (e.g. Ledgerwood and Petry 2010; Steel and Blaszczynski 1996; Suomi et al. 2014). Although this provides support for the emotionally vulnerable group (Blaszczynski and Nower 2002), it does so only in a limited manner; some studies (e.g. Nower et al. 2012; Vachon and Bagby 2009) have found this subgroup to report high levels of impulsivity, personality disorders and hostility, which according to the Pathways Model falls within the antisocial-impulsivist pathway. However, most research has explored *current* levels of negative affect, rather than assessing emotional dysfunction that commenced *prior* to their gambling history, as suggested by the Pathways Model (e.g. Bonnaire et al. 2009; Ledgerwood and Petry 2010; Stewart et al. 2008; Suomi et al. 2014). Finally, several studies have found support for a group of gambling individuals who display marked impulsivity (e.g. Ledgerwood and Petry 2010; Stewart and Zack 2008; Suomi et al. 2014; Turner et al. 2008), thus suggesting some value for the antisocial-impulsivist pathway. Again, however, this is not wholly consistent with the Pathways Model as it does not capture antisocial behaviour and personality, which are core elements of this pathway.

Whilst the Pathways Model was originally conceptualised for problem and pathological gamblers, it was originally intended to identify subgroups applicable to the development of gambling problems *across* the spectrum of disorder (Nower et al. 2012). Nevertheless, there has been little research regarding the Pathways Model in those who gamble regularly *but* have not been identified as pathological/problem gamblers; no studies to date, for example, appear to have examined the model in either a sample of gamblers recruited from online gambling forums or in university students.

The current series of studies aims to address this area, drawing from the Pathways Model to propose a model capturing the different types of gamblers, a model that incorporates factors of relevance to risk but introduces a potential role for protective factors. Such factors are those that could move an individual away from problem gambling. The studies employ samples of students and gambling forum users. This allows for a broader range of participants to be considered, including those who do not meet criteria for problem or pathological gambling. The following overall predictions were made:

1. There will be a cluster of gamblers similar to Blaszczynski and Nower’s (2002) behaviourally conditioned pathway, with lower levels of premorbid and current anxiety and depression, impulsivity, and negative life experiences evidenced.
2. There will be a cluster of gamblers similar to Blaszczynski and Nower’s (2002) emotionally vulnerable pathway, with increased levels of premorbid and current anxiety and depression, negative life events and impulsivity evidenced.
3. There will be a cluster of gamblers similar to Blaszczynski and Nower’s (2002) antisocial-impulsivist pathway, with more impulsivity and offending noted than those within the emotionally vulnerable and behaviourally conditioned groups.
4. Those in a pathway similar to the behaviourally conditioned pathway will report the highest levels of protective factors, with those similar to the antisocial-impulsivist, the least (Dickson et al. 2002, 2008; Lussier et al. 2007).

## Study 1

This study aims to explore the utility of classifying gamblers into groups based on their primary motives for gambling. It explores the levels of anxiety, depression and substance use in each subgroup, to establish if levels approximate to those proposed in the Pathways Model.

## Method

### Participants

Six hundred and ninety-four participants took part, 553 men and 141 women. Two hundred and four participants were students and 490 were online gambling forum users. Regarding students, 77% (*n* = 158) were 18–25 years of age, 13% (*n* = 27) were 26–35, 5% (*n* = 11) were 36–45 years of age and 4% (*n* = 8) were 46–55. Regarding gambling forum users, 29% (*n* = 142) were 18–25, 39% (*n* = 190) were 26–35 years of age, 17% (*n* = 83) were 36–45, 10% (*n* = 51) were 46–55 years of age, and 5% (*n* = 24) were 55 years of age or older. The response rate for completion of the questionnaires was 39%.

### Measures

DSM-5 Diagnostic Criteria for Disordered Gambling (DSM-5: APA 2013). The DSM-5 symptom criteria were operationalised into nine self-report items, for the current study. It focused on the last 12 months (e.g. ‘Have you needed to gamble with increasing amounts of money in order to achieve the desired excitement?’). Each item required a yes/no response. Gambling severity was based on the number of criteria endorsed. It scored participants into one of three categories, based on DSM-5 symptom number; Mild Gambling Disorder (scoring 4–5), Moderate Gambling Disorder (scoring 6–7), and Severe Gambling Disorder (scoring 8–9).

The Problem Gambling Severity Index (PGSI: Ferris and Wynne 2001), comprised of nine items (e.g. ‘How often have you bet more than you could really afford to lose’). The scale is rated and scored on a four-point Likert Scale: (0) never/almost never (1) Sometimes, (2) Most of the time (3) Almost Always. Zero classifies participants as a ‘non-problem gambler’, one to two as a ‘low risk’ gambler, three to seven as ‘moderate risk’ gamblers and eight or more as ‘problem gamblers’. Regarding internal reliability, *α* = .86 and .90 for students and gambling forum users respectively.

Gambling Motives Questionnaire (GMQ: Stewart and Zack 2008), a 15 item measure comprising three subscales: Social Motives, Enhancement Motives, and Coping Motives. Each subscale contained five items rated on a four-point Likert scale: (1) never (2) almost never (3) sometimes, and (4) almost always. Internal reliabilities were as follows; Coping–*α* = .77 for students and .74 for gambling forum users; Social–*α* = .70 for students and .70 for gambling forum users; Enhancement–*α* = .86 for students and .85 for gambling forum users.

Hospital Anxiety and Depression Scale (HADS: Zigmond and Snaith 1983), a 14 item scale capturing anxiety and depression. Seven items comprise the anxiety scale and seven the depression scale. Each of the two subscales were rated on a four point Likert scale: e.g. (1) not at all (2) occasionally (3) quite often, and (4) very often. Internal reliabilities were as follows; Anxiety–*α* = .78 for students and .84 for gambling forum users; Depression–*α* = .78 for students and .82 for gambling forum users.

The Alcohol Use Disorder Identification Test (AUDIT: Babor et al. 2001), a 10 item scale that measures harmful use, abuse and dependence of alcohol. Each of the scale items are rated on a five point Likert scale: (1) Never (2) Less than monthly (3) Monthly (4) Weekly, (5) Daily or almost daily. Regarding internal reliability, *α* = .83 and .81 for students and gambling forum users respectively.

Drug Abuse Screening Test–10 (DAST-10: Skinner 1982), a 10 item brief screening tool that assesses drug use, not including alcohol or tobacco, in the past 12 months. Each item requires a yes/no response (e.g. Do you abuse more than one drug at a time?). Items that are answered yes are allocated a score of one. The higher the score the more severe the drug use.

### Procedure

To recruit student participants, the study was advertised on an online newsletter, across university sites. Posters detailing the research were also placed in various social areas in one university. To recruit those from gambling forums, the study was advertised on three gambling forums. These forums were specifically designed for individuals to discuss gambling. The questionnaires were administered online using Survey Gizmo. No incentive was provided.

## Results

### Primary Gambling Motives

To determine which of the three gambling motives was the primary motive for each participant, the scores of the three gambling motive scales (enhancement [antisocial-impulsivist], social [behaviourally conditioned] and coping [emotionally vulnerable]) were converted into standardised residuals (z-scores) so residuals from the three motives could be compared. The motive with the highest z-score was allocated as the participants’ primary motive for gambling. The mean and standard deviation values for each gambling motive for men, women, students and forum users are presented in Table 1.

**Table 1:** Descriptive statistics for each variable by primary motive, sex, and sample type

	Men			Women			Overall		
Primary motive	Student	Forum user	Total	Student	Forum user	Total	Student	Forum user	Total
Enhancement	*M* (SD) *N* = 35	*M* (SD) *N* = 155	*M* (SD) *N* = 190	*M* (SD) *N* = 8	*M* (SD) *N* = 17	*M* (SD) *N* = 25	M (SD) *N* = 43	*M* (SD) *N* = 172	*M* (SD) *N* = 215
PGSI	4.7 (3.4)	4.5 (3.9)	4.5 (3.8)	3.0 (3.2)	2.8 (3.0)	2.8 (3.0)	4.4 (3.4)	4.3 (3.9)	4.3 (3.8)
Anxiety	4.8 (3.6)	4.6 (3.5)	4.7 (3.5)	5.8 (3.0)	5.0 (2.3)	5.2 (2.5)	5.0 (3.5)	4.7 (3.4)	4.7 (3.4)
Depression	3.0 (3.3)	2.6 (2.8)	2.7 (2.9)	3.6 (4.0)	2.3 (2.3)	2.7 (2.9)	3.1 (3.4)	2.6 (2.8)	2.7 (2.9)
Alcohol	10.1 (7.0)	11.1 (6.4)	10.9 (6.5)	7.0 (6.2)	7.4 (4.3)	7.4 (4.7)	9.6 (6.9)	10.7 (6.4)	10.5 (6.5)
Drugs	0.8 (1.1)	1.2 (1.9)	1.1 (1.8)	1.1 (1.7)	0.5 (1.1)	0.7 (1.3)	0.9 (1.2)	1.1 (1.9)	1.0 (1.7)

Coping	*N* = 51	*N* = 132	*N* = 183	*N* = 16	*N* = 21	*N* = 37	*N* = 67	*N* = 153	*N* = 220
PGSI	5.5 (5.0)	6.5 (5.7)	6.2 (5.3)	2.9 (4.8)	5.1 (6.0)	4.1 (5.5)	4.9 (5.1)	6.3 (5.8)	5.9 (5.6)
Anxiety	7.5 (4.9)	7.1 (4.70)	7.2 (4.7)	8.0 (4.4)	9.3 (4.1)	8.7 (4.3)	7.6 (4.7)	7.4 (4.7)	7.4 (4.7)
Depression	4.9 (4.3)	5.1 (4.1)	5.0 (4.2)	3.9 (2.6)	6.9 (4.4)	5.6 (4.0)	4.7 (3.9)	5.3 (4.2)	5.1 (4.1)
Alcohol	10.7 (8.0)	10.2 (7.1)	10.4 (7.3)	6.7 (5.1)	8.6 (6.7)	7.7 (6.1)	9.7 (7.6)	10.0 (7.1)	9.9 (7.2)
Drug	1.6 (2.8)	1.1 (1.9)	1.2 (2.2)	1.2 (2.3)	1.2 (2.2)	1.2 (2.2)	1.5 (2.7)	1.1 (2.0)	0.9 (1.5)

Social	*N* = 55	*N* = 125	*N* = 180	*N* = 39	*N* = 40	*N* = 79	*N* = 94	*N* = 165	*N* = 259
PGSI	3.1 (3.3)	3.2 (3.5)	3.1 (3.4)	1.6 (1.8)	1.1 (1.6)	1.4 (1.7)	2.5 (2.8)	2.7 (3.2)	2.6 (3.1)
Anxiety	5.5 (3.7)	4.5 (3.3)	4.8 (3.5)	7.05 (3.8)	5.5 (3.4)	6.3 (3.7)	6.2 (3.8)	4.7 (3.4)	5.2 (3.6)
Depression	2.7 (2.7)	2.7 (3.0)	2.7 (8.9)	3.8 (3.2)	3.4 (3.0)	3.6 (3.1)	3.2 (2.9)	2.9 (3.0)	3.0 (3.0)
Alcohol	10.7 (5.8)	9.9 (5.3)	10.1 (5.4)	11.1 (6.1)	6.7 (4.3)	9.0 (5.7)	10.9 (5.9)	9.1 (5.2)	9.8 (5.5)
Drug	0.7 (1.2)	1.1 (1.6)	0.9 (1.5)	0.9 (1.8)	0.5 (0.8)	0.7 (1.4)	0.7 (1.5)	0.9 (1.5)	0.8 (1.5)

### Primary Gambling Motives, Gambling Severity and their Association With Psychopathology

To determine whether psychological distress (anxiety and depression) and substance misuse (alcohol and drugs) were predictive of gambling severity in those with primary social, coping and enhancement motives, a series of hierarchical multiple regressions were performed with each primary motive as an interaction term. This consisted of three hierarchical regressions for each primary motive. The first regression (Regression 1) for each motive included the motive of interest as an interaction term for anxiety, depression, drug use and alcohol use. The interaction term, if significant, showed a significant difference between those with that particular motive as their primary gambling motive and those without that motive as their primary gambling motive, for each predictor variable. The second hierarchical regression (Regression 2) in each series included only data from individuals who *had* that specific gambling motive as their primary gambling motive. The third regression (Regression 3) in each series included only those who *did not have* that specific motive as their primary gambling motive. Sex and sample type were entered as predictor variables in each regression in step 1 to control for any possible effects. Anxiety, depression, alcohol and drug use were entered in each regression in step 2.

### Primary Gambling Motive: Social

The first regression (Regression 1) was significant on step 1, *F* (2, 669) = 14.5, MSE = 18.8, *p *< .001, and step 2, *F* (11, 660) = 32.8, MSE = 12.9, *p *< .001. The proportion of variance explained by the whole model was 34%. Anxiety emerged as a significant interaction term. The next regression (Regression 2), for participants who have a primary social motive for gambling, was significant on step 1: *F* (2, 250) = 9.10, MSE = 9.05, *p* = < .001, and on step 2, *F* (6, 246) = 11.71, MSE = 7.68, *p *< .001. The proportion of variance explained by the whole model was 20%. Increased depression, alcohol use, and drug use emerged as significant predictors of gambling severity. The beta values indicate that depression was the most predictive (.27), followed by drug use (.17) and alcohol use (.14). The next regression (Regression 3), for those without a primary social motive, was significant on step 1, *F* (2, 416) = 3.10, MSE = 23.02, *p* = .046, and on step 2, *F* (6, 412) = 32.67, MSE = 15.99, *p *< .001. The proportion of variance explained by the model was 31% and increased anxiety, depression and alcohol use were significantly predictive of gambling severity. The beta values indicated that anxiety (.32) was the most predictive, followed by depression (.20) and drug use (.17). The interaction term, anxiety, was significantly more predictive of gambling severity for the non-primary social gamblers.

### Primary Gambling Motive: Coping

This replicated the approach to regression as per the prior motive. The initial regression (Regression 1) was significant on step 1, *F* (2, 669) = 14.5, MSE = 18.8, *p *< .001, and step 2, *F* (11, 660) = 31.9, MSE = 13.5, *p *< .001. The proportion of variance explained by the model was 34%. Anxiety and drug use emerged as significant interactions; showing significant differences between those with primary coping motives and those without. The subsequent regression for those with a primary coping motive (Regression 2) emerged as significant on step 1, *F* (2, 209) = 3.1, MSE = 30.4, *p* = .046, and on step 2, *F* (6, 205) = 21.8, MSE = 19.5, *p *< .001. The proportion of variance explained by the model was 37%, with increased anxiety, depression, and alcohol use emerging as significantly predictive of gambling severity. The beta values indicated that anxiety (.34) was the most predictive, followed by depression (.22) and alcohol use (.21). The interaction term, anxiety, was significantly more predictive of gambling severity in this group than in those without a primary coping motive. For those without primary coping as a motive (Regression 3), the regression emerged as significant on step 1, *F* (2, 457) = 14.5, MSE = 11.7, *p *< .001, and on step 2, *F* (6, 453) = 19.3, MSE = 10.03, *p *< .001. The proportion of variance explained by the model was 19%. Increased anxiety, depression and alcohol use emerged as significant predictors of gambling severity. However, the beta values indicated that, in this group, anxiety (.15), depression (.14) and alcohol use (.14) were less predictive than in those *with* primary coping motives for gambling. Drug use was more predictive of gambling severity than in those with primary coping motives for gambling.

### Primary Gambling Motive: Enhancement

The first regression (Regression 1) was significant on step 1, *F* (2, 669) = 14.5, MSE = 18.8, p < .001, and step 2, *F* (11, 660) = 28.4, MSE = 13.5, *p *< .001. The proportion of variance explained by the model was 31%. Depression emerged as an interaction term, showing a significant difference between those with a primary enhancement motive and those without. For those with a primary enhancement motive (Regression 2), the regression emerged as non-significant on step 1, *F* (2, 204) = 1.8, MSE = 14.2 ns. However, it was significant on step 2, *F* (6, *200*) = 7.8, MSE = 11.9, *p *< .001. The proportion of variance explained by the model was 17%. In this group, increased anxiety emerged as significant predictor of gambling severity. For those without a primary enhancement motive (Regression 3) the regression emerged as significant on step 1, *F* (2, 462) = 12.4, MSE = 20.2, *p *< .001, and on step 2, *F* (6, 458) = 43.7, MSE = 14.1, *p *< .001. The proportion of variance explained by the model was 36%. In this group, increased anxiety, depression and alcohol use emerged as significantly predictive of gambling severity.

## Discussion

This study provided some evidence for the Pathways towards Problem and Pathological Gambling Model (Blaszczynski and Nower, 2002) regarding subtyping gamblers into distinct groups. It placed individuals into subgroups based on their primary motivation to gamble. In primary social gamblers, increased depression, drug and alcohol use were predictive of gambling severity, whereas anxiety was less predictive of gambling severity than in those who gambled for coping and enhancement purposes. Those who gambled primarily to cope displayed the most severe levels of psychological distress. Anxiety was more predictive of gambling severity in this group in comparison to those who gambled primarily for social and enhancement reasons. Those who gamble primarily for enhancement purposes, reported less depression than those with primary social and coping gambling motives.

Despite some consistencies with the Pathways Model, the current research does not fully support it. For example, the current study explored the emotionally vulnerable pathway for individuals who had a primary coping motive for gambling *and* the antisocial-impulsivist pathway for those with a primary enhancement motive. According to the Pathways Model, coping and enhancement gamblers could theoretically both appear in the emotionally vulnerable pathway. This includes those who gamble to cope with negative affect *and* to enhance positive affect. Therefore, whilst enhancement motives alone may not have been adequate to explore the antisocial-impulsivist pathway, different levels of anxiety, depression, drug and alcohol use were found in individuals with primary coping and enhancement motives. In addition, the comorbidities noted here were differentially predictive of problem gambling in the primary coping and enhancement gamblers. This suggests that these two types of gamblers represent different subgroups of gamblers. That is, those who gamble to reduce negative affect and those who gamble to enhance positive affect are not solely in one ‘emotionally vulnerable’ pathway.

Overall, it was apparent there was no single factor contributing to the development and maintenance of problematic gambling. The findings provided some support to Blaszczynski and Nower’s (2002) Pathways Model. This was through identifying subgroups of gamblers with different etiological and clinical characteristics, somewhat comparable to the behaviourally conditioned, emotionally vulnerable and antisocial-impulsivist pathways (as identified by primary social, coping and enhancement gambling motivations). However, to fully explore this model, further research is required. This was addressed in Study 2.

## Study 2

Study two builds on the earlier study by assessing in more detail the behaviourally conditioned and emotionally vulnerable pathways. It extends variables considered to include gambling beliefs, association with peers who gamble, current and premorbid psychological distress, negative life events and impulsivity. It explores whether there is a group of gamblers similar to the behaviourally conditioned and emotionally vulnerable pathways in Blaszczynski and Nower’s (2002) model.

## Method

### Participants

Six hundred and sixty-nine participants took part; 63% (*n* = 421) were men and 37% (*n* = 248) were women. Of these, 265 were students and 404 gambling forum users. Regarding student participants, 77% (*n* = 205) were 18–25 years of age, 16% (*n* = 43) were 26–35 years of age, 4% (*n* = 10) were 36–45 years of age, 2% (*n* = 6) were 46–55 years of age, and 0.4% (*n* = 1) were 55 years of age or older. Regarding gambling forum users, 24% (*n* = 98) were 18–25 years of age, 37% (*n* = 150) were 26–35 years of age, 18% (*n* = 72) were 36–45 years of age, 14% (*n* = 56) were 46–55 years of age, and 7% (*n* = 27) were 55 years of age or older. Ninety-six percent identified as a recreational gambler and 4% as a professional gambler. The response rate for completion was 35%.

### Measures

DSM-5 Diagnostic Criteria for Disordered Gambling and The Problem Gambling Severity Index (PGSI), as for Study 1. Regarding PGSI internal reliability, *α* = .91 for both samples. The Hospital Anxiety and Depression Scale (HADS) was also applied but here was presented twice to participants, once for symptoms experienced in the past couple of weeks (C-HADS), and again in relation to symptoms prior to engagement in gambling (named P-HADS). Regarding internal reliabilities, this was as follows: C-HADs; Anxiety–*α* = .87, Depression *α* = .75, for both groups; P-HADs; Anxiety–*α* = .88 for students and *α* = .90 for gambling forum users; Depression–*α* = .79 for students and *α* = .84 for gambling forum users.

There were four measures unique to the current study, as follows:

Gamblers’ Beliefs Questionnaire (GBQ: Steenbergh et al. 2002), a 21 item scale measuring gambling-related cognitive distortions, for all forms of gambling. The scale is rated and scored on a five-point Likert Scale: (1) Strongly Disagree, (2) Disagree, (3) Neutral, (4) Agree, (5) Strongly Agree. The scale authors reported two factors within the scale: luck/perseverance and illusion of control. A high score on the GBQ indicates a high level of cognitive distortions. Internal reliability was *α* = .89 for both samples.

The Barratt Impulsivity Scale: Short Version (BIS-15: Spinella 2007), a 15 item scale measuring non-planning impulsivity, motor impulsivity, and attentional impulsivity. It is rated on a four-point Likert Scale: (1) Rarely/Never, (2) Occasionally, (3) Often, (4) Almost Always/Always. Higher scores indicate increased levels of impulsivity. Internal reliability was *α* = .84 for both samples.

Negative Life Events Scale, a four item scale designed for the current research to measure previous negative life experiences (e.g. ‘I have had many bad things happen to me’). The scale is rated and scored on a four-point Likert Scale: (0) Does not apply, (1) Applies a bit, (2) Applies quite a lot, (3) Totally applies. A higher score indicates increased levels of negative life experiences. Internal reliability was *α* = .74 for students and .75 for gambling forum users.

Gambling Associates Scale, designed for the current study to measure the extent to which participants’ parents, friends, family, and colleagues gamble (e.g. ‘How often do your friends gamble?’). The scale is rated and scored on a five-point Likert Scale: (0) I don’t know, (1) Never, (2) Sometimes, (3) Most of the time, (4) Always. Higher scores indicate increased levels of associates who gamble. It is not a psychometric.

### Procedure

The procedure was as for Study 1.

## Results

This section will present a cluster analysis on the measures included, followed by the creation of groups based on premorbid anxiety and depression. These will be formed to distinguish subgroups, similar to the behaviourally conditioned and emotionally vulnerable groups. The groups are then compared to establish differences within the pathways.

### Exploring Clusters of Gamblers

To explore for homogenous subgroups within the data set, a two-step cluster analysis was performed on the current and premorbid anxiety and depression scales, gambling beliefs, impulsivity, negative life events and gambling associates scales. This was conducted on the entire dataset as there were no population differences (i.e. student versus gambling forum) in relation to gambling severity (*F*[1, 664] = 2.8 ns), with the PGSI scale not entered as a variable in the formation of the clusters. Instead, it was entered as an evaluation variable to allow for exploration of differences between the clusters in the levels of gambling severity reported.

The analysis yielded two clusters based on Schwarz’s BIC and the Log-likelihood distance measures (ratio of distances measures = 3.52). Cluster 1, ‘low comorbidity’, represented 42.8% (*n *= 286) of the sample; Cluster 2, ‘high comorbidity’, represented 57.2% (*n *= 383) of the sample. The level of importance of the variables in determining the clusters demonstrated that premorbid anxiety and depression, followed by current anxiety and depression, and negative life events, showed the largest importance in forming and distinguishing clusters. Descriptive statistics for each measure, in both clusters, were also computed, with significant differences noted. These are presented in Table 2.

**Table 2:** Cluster Profiles: Mean and SD values for each measure for both clusters and for each cluster

Variables	Low comorbidity (Cluster A)					High comorbidity (Cluster B)					
	Men (*n* = 254)	Women (*n* = 129)	Students (*n* = 125)	Forum users (*n* = 258)	Total (*n* = 383)	Men (*n* = 167)	Women (*n* = 119)	Students (*n* = 140)	Forum users (*n* = 146)	Total (*n* = 286)	Total sample (*n* = 669)
	*M* (SD)	*M* (SD)	*M* (SD)	*M* (SD)	*M* (SD)	*M* (SD)	*M* (SD)	*M* (SD)	*M* (SD)	*M* (SD)	*M* (SD)
P-anxiety	2.8 (2.5)	2.8 (2.6)	2.9 (2.5)	2.8 (2.5)	2.8 (2.5)	8.7 (4.0)	10.5 (3.9)	9.5 (4.2)	9.4 (3.9)	9.4 (4.1)	5.7 (4.6)*
P-depression	1.2 (1.3)	1.1 (1.3)	1.2 (1.3)	1.2 (1.3)	1.2 (1.3)	5.8 (3.4)	5.8 (3.6)	5.6 (3.4)	5.9 (3.5)	5.8 (3.5)	3.1 (3.4)*
C-anxiety	3.4 (2.5)	3.4 (2.8)	3.7 (2.8)	3.2 (2.4)	3.4 (2.6)	9.0 (3.8)	10.3 (3.7)	10.0 (3.9)	9.0 (3.7)	9.5 (3.9)	6.0 (4.4)*
C-depression	1.6 (1.5)	1.5 (1.6)	1.5 (1.4)	1.5 (1.6)	1.5 (1.6)	5.6 (2.9)	5.9 (3.2)	5.9 (3.1)	5.6 (3.0)	5.7 (3.1)	3.3 (3.1)*
N. L. E	2.7 (1.9)	3.1 (2.3)	2.6 (2.0)	3.0 (2.0)	2.9 (2.0)	5.1 (2.1)	5.3 (2.5)	5.1 (2.2)	5.2 (2.3)	5.1 (2.3)	3.8 (2.4)*
G. A	7.5 (1.5)	7.3 (1.6)	7.3 (1.7)	7.5 (1.5)	7.4 (1.5)	7.4 (1.5)	7.4 (1.5)	7.3 (1.5)	7.5 (1.5)	7.4 (1.5)	7.4 (1.5) ns
Impulsivity total	29.9 (5.7)	28.3 (6.5)	29.6 (6.4)	29.2 (5.8)	29.4 (6.0)	35.5 (6.5)	35.7 (6.7)	35.4 (6.5)	35.8 (6.6)	35.6 (6.6)	32.0 (7.0)*
N. P. impulsivity	10.7 (3.2)	9.9 (3.2)	10.3 (3.2)	10.5 (3.3)	10.4 (3.3)	13.5 (3.6)	12.6 (3.4)	12.5 (3.1)	13.8 (3.9)	13.1 (3.6)	11.6 (3.7)*
M. impulsivity	10.0 (2.4)	10.0 (2.6)	10.0 (2.7)	10.0 (2.4)	10.0 (2.5)	11.2 (3.1)	11.8 (3.1)	11.6 (3.3)	11.3 (3.0)	11.4 (3.1)	10.6 (2.9)*
A. impulsivity	8.9 (2.4)	8.2 (2.4)	9.0 (2.6)	8.5 (2.3)	8.7 (2.4)	10.9 (2.6)	11.3 (2.9)	11.4 (2.6)	10.8 (2.8)	11.1 (2.7)	9.7 (2.9)*
Gambling beliefs	52.2 (11.0)	39.4 (11.4)	50.3 (13.5)	46.7 (12.1)	47.9 (12.7)	56.3 (11.6)	46.3 (12.2)	53.1 (12.1)	51.2 (13.5)	52.1 (12.8)	49.7 (12.9)*
Illusion of Control	24.0 (5.4)	16.9 (5.4)	22.2 (6.7)	21.3 (6.2)	21.6 (6.4)	23.5 (5.5)	19.7 (5.2)	22.4 (5.7)	21.6 (5.7)	22.0 (5.7)	21.7 (6.1) ns
Luck/Perseverance	28.5 (7.1)	22.0 (7.1)	28.1 (8.1)	25.5 (7.4)	26.3 (7.7)	33.0 (8.3)	26.6 (8.9)	30.9 (8.5)	29.9 (9.6)	30.4 (9.1)	28.1 (8.6)*
Evaluation variable											
PGSI	2.9 (3.1)	1.1 (2.6)	2.5 (3.5)	2.1 (2.9)	2.3 (3.1)	6.7 (4.9)	2.6 (3.8)	4.7 (5.0)	5.2 (4.9)	5.0 (4.9)	3.4 (4.2)*

NB: Two cluster solution: Schwarz’s BIC 3084.81; BIC Change -725.00; Ratio of BIC changes 1.00; Ratio of distance measures 3.53; P-anxiety and P-depression = symptoms from the HADS experienced prior to engagement in gambling; C-anxiety and C-depression = symptoms from the HADS experienced in the past couple of weeks (C-HADS); N.L.E = Negative Life Events; G. A = gambling associates; N. P impulsivity = Non-planning impulsivity; M. impulsivity = Motor Impulsivity; A. impulsivity = Attentional impulsivity. *Significant difference between the two clusters at *p *< .001 level; ns = no significant difference between the two clusters

A MANCOVA was performed to compare differences between the low and high comorbidity clusters on gambling severity using the PGSI and DSM-5 as the dependant variables, with sex as a covariate. There was a significant effect of cluster type on gambling severity, *F* (2, 648) = 49.54, *p *< .001; Wilk’s Λ = 0.87, partial η^2^ = .13, with a significant effect of cluster type on both the PGSI, *F* (1, 649) = 97.62, *p *< .001, partial η^2^ = .13, and the DSM-5, *F* (1, 649) = 79.50, *p *< .001, partial η^2^ = .11. Those in the high comorbidity cluster reported significantly higher levels of gambling than those in the low comorbidity cluster.

### Testing Gambling Groups

To explore and differentiate between the behaviourally conditioned and emotionally vulnerable pathways, groups were formed based on scores on premorbid anxiety and depression scales. Groups were formed using these scores due to them showing the greatest importance in differentiating between clusters. In addition, these are variables that specifically differentiate between the behaviourally conditioned and emotionally vulnerable pathways in Blaszczynski and Nower’s (2002) model.

The premorbid anxiety and depression scales were split into ‘low’ and ‘high’ based on their scale score; participants were categorised into ‘high’ or ‘low’ on the anxiety and depression scale if they scored higher than the scale cut off point of > 8. This was the cut-off point from normal to mild anxiety/depression proposed by the scale authors and have been applied widely in the literature since. Individuals categorised as high on either scale were assigned to the emotionally vulnerable group. The groups were then used to explore differences on the measures. Once the behaviourally conditioned and emotionally vulnerable groups were established, descriptive statistics on each measure were calculated. These are presented in Table 3.

**Table 3:** The Mean and SD values for each variable in total and split by group and sex

Variables	Behaviourally conditioned					Emotionally vulnerable				
	Men (*n *= 301)	Women (*n* = 149)	Students (*n* = 158)	Forum users (*n* = 292)	Total (*n* = 450)	Men (*n* = 120)	Women (*n* = 99)	Students (*n* = 107)	Forum users (*n* = 112)	Total (*n* = 219)
	*M* (SD)	*M* (SD)	*M* (SD)	*M* (SD)	*M* (SD)	*M* (SD)	*M* (SD)	*M* (SD)	*M* (SD)	*M* (SD)
PGSI	3.5 (3.8)	1.2 (2.8)	3.1 (4.1)	2.6 (3.3)	2.7 (3.6)	6.7 (4.8)	2.7 (4.0)	4.6 (4.8)	5.1 (4.9)	4.9 (4.9)
DSM-5	1.9 (2.4)	0.6 (1.4)	1.7 (2.4)	1.4 (2.1)	1.5 (2.2)	3.7 (3.0)	1.3 (2.1)	2.3 (2.6)	2.8 (3.2)	2.6 (2.9)
GBQ total	52.5 (10.9)	40.5 (11.7)	50.6 (12.9)	47.5 (12.2)	48.6 (12.5)	57.0 (12.0)	46.0 (12.5)	53.5 (12.6)	50.7 (14.0)	52.1 (13.4)
Illusion of control	23.7 (5.3)	17.3 (5.5)	22.1 (6.3)	21.3 (6.1)	21.6 (6.2)	24.1 (5.8)	19.6 (5.1)	22.5 (6.0)	21.7 (5.9)	22.1 (5.9)
Luck/perseverance	29.1 (7.2)	22.6 (7.7)	28.5 (8.1)	26.2 (7.7)	27.0 (7.9)	33.2 (8.6)	26.6 (9.1)	31.2 (8.6)	29.3 (10.1)	30.3 (9.4)
C-anxiety	4.2 (3.2)	4.3 (3.5)	4.9 (3.8)	3.8 (2.9)	4.2 (3.3)	9.1 (4.1)	10.3 (4.0)	10.1 (4.1)	9.2 (4.0)	9.6 (4.1)
C-depression	2.3 (2.3)	2.2 (2.6)	2.4 (2.4)	2.2 (2.3)	2.3 (2.4)	5.4 (3.3)	5.7 (3.3)	5.9 (3.3)	5.2 (3.2)	5.5 (3.3)
N.L.E	3.2 (2.1)	3.5 (2.4)	3.1 (2.3)	3.4 (2.20	3.3 (2.2)	4.9 (2.3)	5.2 (2.5)	5.1 (2.3)	4.9 (2.5)	5.0 (2.4)
Impulsivity total	31.0 (6.5)	29.3 (7.2)	31.3 (7.3)	30.0 (6.5)	30.4 (6.8)	34.9 (6.2)	35.7 (6.3)	34.7 (6.2)	35.7 (6.2)	35.3 (6.3)
N.P impulsivity	11.3 (3.6)	10.2 (3.3)	10.9 (3.4)	11.0 (3.6)	10.9 (3.5)	13.0 (3.6)	12.8 (3.4)	12.1 (3.0)	13.7 (3.8)	12.9 (3.5)
M impulsivity	10.3 (2.6)	10.3 (2.9)	10.6 (3.2)	10.1 (2.5)	10.2 (2.7)	11.0 (3.0)	11.6 (3.0)	11.1 (3.1)	11.5 (2.9)	11.3 (3.0)
A impulsivity	9.2 (2.5)	8.6 (2.8)	9.5 (2.8)	8.7 (2.5)	9.0 (2.6)	11.0 (2.7)	11.4 (2.7)	11.4 (2.7)	10.9 (2.8)	11.2 (2.7)
G. A	7.4 (1.5)	7.3 (1.6)	7.2 (1.7)	7.4 (1.5)	7.3 (1.6)	7.5 (1.5)	7.5 (1.5)	7.3 (1.5)	7.8 (1.4)	7.5 (1.5)

*PGSI* problem gambling severity index, *DSM*-*5* diagnostic and statistical manual- fifth edition, *GBQ* gambling beliefs questionnaire, *N.L.E.* negative life events., *N. P impulsivity* non-planning impulsivity; *M. impulsivity* motor impulsivity, *A. impulsivity* attentional impulsivity, *G. A* gambling associates

There was a significant effect of group on current psychological distress, *F* (2, 663) = 170.0, *p *< .001; Wilk’s Λ = 0.66, partial η^2^ = .34. Significant main effects were found for group on anxiety levels, *F* (1664) = 306.0, *p *< .001, partial η^2^ = .32, and depression levels, *F* (1664) = 203.6, *p *< .001, partial η^2^ = .24, with the emotionally vulnerable group reporting significantly higher levels of current anxiety and depression than the behaviourally conditioned group. There was a significant effect of group on impulsivity, *F* (1664) = 76.95, *p *< .001, with the emotionally vulnerable pathway reporting higher levels of impulsivity than the behaviourally conditioned pathway. There was a significant effect of group on levels of cognitive distortions, after controlling for the effect of sex and sample type, *F* (1664) = 22.0, *p *< .001, with the emotionally vulnerable pathway reporting significantly higher levels of gambling related cognitive distortions than the behaviourally conditioned pathway. There was a significant effect of group on self-reported negative life experiences, *F* (1664) = 81.4, *p *< .001, with the emotionally vulnerable pathway reporting higher levels than the behaviourally conditioned pathway. There was no effect of group regarding self-reported gambling associates, *F* (1664) = 3.45 ns.

## Discussion

Two distinct groups of gamblers emerged. The first resembled the primary social subgroup identified in Study 1. It comprised individuals who scored low on pre-existing and current anxiety and depression, impulsivity, negative life events and gambling related cognitive distortions. The second cluster reported higher scores on each of the identified variables. The behaviourally conditioned and emotionally vulnerable subgroups constructed were distinguished by self-reported levels of premorbid anxiety and/or depression. This was a key feature of the emotionally vulnerable subgroup proposed by Blaszczynski and Nower (2002). Similar to the Pathways Model, the findings here demonstrated that premorbid psychological distress emerged as the key variable in differentiating the clusters. This provides direct support for the emotionally vulnerable pathway, who are described as distinct from the behaviourally conditioned pathway through their premorbid affective disturbances. The current research does not support studies, however, that have failed to find a gambler subgroup *without* severe psychopathology (e.g. Ledgerwood and Petry 2006). Increasing studies are finding such a group, which provides strong support for the presence of a type gambler absent of psychopathology and who primarily gambles for social purposes (Stewart and Zack 2008).

Nevertheless, the findings provided evidence for identifiable clinical groups of student and forum user gamblers, which supports major tenets of the Pathways Model (Blaszczynski and Nower 2002). That is, there is a clear subgroup of gamblers devoid of severe psychopathology, cognitive distortions, and low levels of gambling involvement (Blaszczynski and Nower 2002; Ledgerwood and Petry 2010; Lesieur 2001; Stewart and Zack 2008; Stewart et al. 2008; Turner et al. 2008; Suomi et al. 2014; Vachon and Bagby 2009). There is also a pathway characterised by significant affective instability (Blaszczynski and Nower 2002; Ledgerwood and Petry 2010; Lesieur and Blume 1991; Steel and Blaszczynski 1996; Stewart and Zack 2008; Suomi et al. 2014; Turner et al. 2008).

Unique to the current study is the testing of the behaviourally conditioned pathway. This was conducted by exploring gambling beliefs and participants’ friends and family who gamble, rather than relying on an ‘absence’ of other features to test this pathway. Furthermore, many studies have identified a subgroup of gambler with significant emotional vulnerabilities (e.g. Ledgerwood and Petry 2010; Lesieur and Blume 1991; Steel and Blaszczynski 1996; Stewart and Zack 2008; Suomi et al. 2014; Turner et al. 2008), with the current study extending this by finding a group characterised by pre-existing affective vulnerabilities and negative life events, alongside severe current affective disorders and increased cognitive distortions. However, a third pathway, antisocial-impulsivist, which is key to the Pathways Model, was not specifically examined in the current study, but is required (Blaszczynski and Nower 2002). This is a clear limitation and will be focused in the ensuing study. Furthermore, whilst the Pathways Model proposes subgroups of gamblers incorporating different gambling related risk factors, it completely neglects protective factors, which could lead an individual away from problem gambling (Dickson et al. 2002, 2008; Lussier et al. 2007). The ensuing study will therefore consider factors associated with the antisocial-impulsivist subgroup of gamblers, including impulsivity, psychopathy and offending behaviour and extend it to capture protective factors. This will allow for a more comprehensive exploration of all three pathways in Blaszczynski and Nower’s (2002) pathways model to be obtained.

## Study 3

This final study explores the risk factors associated with the antisocial-impulsivist pathway (Blaszczynski and Nower 2002), which have not been sufficiently explored in prior studies. This includes antisocial personality, offending behaviour and impulsivity. It further explores protective factors within each of the gambler subgroups.

### Participants

Five hundred and seventy-nine participants took part; 71% (*n* = 413) were men and 29% (*n* = 166) were women. Two hundred and one participants were students, 378 were gambling forum users. Regarding student participants, 81% (*n* = 163) were 18–25 years of age, 12% (*n* = 25) were 26–35 years of age, and 7% (*n* = 13) were 36–45 years of age. No students were older than aged 45. Regarding the gambling forum user sample, 19% (*n* = 73) were 18–25 years of age, 34% (*n* = 127) were 26–35 years of age, 21% (*n* = 78) were 36–45 years of age, 16% (*n* = 61) were 46–55 years of age, and 10% (*n* = 39) were 55 years of age or older.

Twenty-two percent of participants (*n* = 127) reported being unemployed, 21% (*n* = 119) were in part-time employment, and 58% (*n* = 333) reported being in full-time employment. The majority of the sample described themselves as White British ethnic origin (79.4%, *n* = 460) and the remainder as White Irish (7.3%, *n* = 42), White other (7.1%, *n* = 41), Asian (1.2%, *n* = 7), Black Caribbean (0.5%, *n* = 3), Black African (0.7%, *n* = 4), Mixed ethnic origin (2.7%, *n* = 16), and Other ethnic origin (0.2%, *n* = 1). Five preferred not to report their ethnic origin.

### Measures

The Problem Gambling Severity Index (PGSI: internal reliability, *α* = .89 for students and .92 for gambling forum users), Barratt Impulsivity Scale – Short Version (BIS-15: internal reliability, *α* = .82 for students and .84 for gambling forum users) and Hospital Anxiety and Depression Scale (HADS, internal reliability: Anxiety *α* = .79 for students and .81 for gambling forum users; Depression *α* = .74 for students and .73 for gambling forum users) were used, as for the previous studies.

In addition, the Psychopathic Processing and Personality Assessment (PAPA-2: Lewis et al. 2017) was employed. This is a 28 item measure. Example items are ‘I am only interested in myself’ and ‘If I am caught out on a lie I can quickly think of a way out’. The scale is rated and scored on a five-point Likert Scale: (1) Very unlike me, (2) Not really like me, (3) Neither agree or disagree, (4) Somewhat like me, (5) Very like me. Internal reliability was at *α* = .88 for students and .89 for gambling forum users.

The following protective measures were used, as follows:

The Satisfaction with Life Scale (Diener et al. 1985), a five-item measure of the satisfaction with life (e.g. ‘In most ways my life is close to my ideal’). The scale is rated on a seven-point Likert Scale: (1) Strongly disagree, (2) Disagree, (3) Slightly disagree, (4) Neither agree nor disagree, (5) Slightly agree, (6) Agree, (7) Strongly agree. It classifies participants as: Extremely satisfied, Satisfied, Slightly satisfied, Neutral, Slightly dissatisfied, Dissatisfied, Extremely dissatisfied. Internal reliability was at *α* = .89 for both groups.

Multidimensional Scale of Perceived Social Support (Zimet et al. 1988), a 12-item scale that measures social support. An example item is ‘I get the emotional help and support I need from my family’. The scale is rated on a seven-point Likert Scale: (1) Very Strongly Disagree, (2) Strongly Disagree, (3) Mildly Disagree, (4) Neutral, (5) Mildly Agree, (6) Strongly Agree, (7) Very Strongly Agree. Internal reliability was at *α* = .91 for students and .94 for gambling forum users.

The Brief Self-Control Scale (BSCS; Tangney et al. 2004), a 13-item measure of self-control. An example item is ‘I say inappropriate things’. The scale is rated and scored on a five-point Likert Scale: (1) Not at all like me, (2) A little like me, (3) Somewhat like me, (4) Mostly like me, (5) Very much like me. Internal reliability was at *α* = .84 for students and .86 for gambling forum users.

The Brief Resilience Scale (Smith et al. 2008), a six item measure of resilience, comprising items such as ‘I usually come through difficult times with little trouble’. The scale is rated on a five-point Likert Scale: (1) Strongly Disagree, (2) Disagree, (3) Neutral, (4) Agree, (5) Strongly Agree. Internal reliability was at *α* = .89 for students and .87 for gambling forum users.

Offending Behaviour History, designed for the current study to examine the extent of offending behaviour. It examined whether participants had committed a violent, acquisitive, drug-related or other antisocial behaviour offence. An example question is ‘Whether you have been convicted or not, have you ever committed a violent offence’. The scale response format required a response of either yes/no.

### Procedure

As for the prior studies.

## Results

The analysis created further subgroups, based on those suggested in Blaszczynski and Nower’s (2002) Pathways Model. It focused on the antisocial-impulsivist group, before exploring evidence for protective factors across all three groups.

### Gambler Group and Exploring for Differences Between Groups

To explore and differentiate between the behaviourally conditioned, emotionally vulnerable, and antisocial-impulsivist pathways, groups were formed based on premorbid anxiety, depression and impulsivity scores. Key factors that differentiated the emotionally vulnerable from the behaviourally conditioned subgroups in Study 2 represented the presence of pre-existing depression and/or anxiety. Thus, the current study used a similar method to create groups based on those suggested in the Pathways Model (Blaszczynski and Nower 2002). The premorbid anxiety and depression scales were each split into ‘low’ and ‘high’ groups. A score lower than the scale cut off point (eight) was classified into the ‘low’ group and those at, or above, this value classified into the ‘high’ anxiety and/or depression group. A median split was performed on the impulsivity scale to separate participants into ‘high’ and ‘low’ levels of impulsivity. The median was 31. Those scoring above were classified into the ‘high’ group and those at or below this value into the ‘low’ group. Individuals categorised as low on anxiety, depression and impulsivity were assigned to the behaviourally conditioned subgroup. Those who scored high on either anxiety or depression and low on impulsivity were assigned to the emotionally vulnerable group. Those who scored high on impulsivity and either anxiety or depression were assigned to the antisocial-impulsivist subgroup. Descriptive statistics are presented in Table 4.

**Table 4:** Descriptive statistics for each of the measures, and offending behaviour, split by group and sex

Measures	Behaviourally conditioned						Emotionally vulnerable						Antisocial impulsivist					
	Men *N* = 250		Women *N* = 83		Total *N* = 333		Men *N* = 57		Women *N* = 29		Total *N* = 86		Men *N* = 104		Women *N* = 50		Total *N* = 154	
	M	SD	M	SD	M	SD	M	SD	M	SD	M	SD	M	SD	M	SD	M	SD
PGSI	3.2	3.9	1.6	3.1	1.9	3.2	3.7	2.8	2.6	4.2	3.3	3.4	6.8	5.7	4.1	4.9	5.9	5.6
BIS-15	29.8	6.2	30.8	6.9	30.0	6.4	25.9	3.5	27.3	2.3	26.4	3.2	37.3	5.1	38.5	5.8	57.7	5.4
Anxiety	3.8	2.0	3.7	2.4	3.8	2.1	9.0	3.8	11.1	2.4	9.7	3.5	10.7	3.1	11.6	3.0	11.0	3.1
Depression	3.2	2.1	2.5	2.1	3.0	2.1	6.7	2.9	5.6	3.1	6.3	3.0	6.8	3.1	6.4	3.4	6.7	3.2
PAPA	61.5	13.3	52.7	14.9	59.3	14.2	66.1	13.2	57.7	14.5	63.3	14.2	75.2	16.9	69.1	14.6	73.2	16.5
*PAPA subscales*																		
DT	15.6	4.7	13.4	5.1	15.1	4.9	14.4	4.4	13.2	3.3	14.0	4.1	19.7	6.4	18.6	6.0	19.3	6.3
ED	9.4	3.4	7.9	3.4	9.0	3.5	9.8	3.5	8.1	4.1	9.2	3.7	10.9	3.3	9.8	3.4	10.6	3.4
DfO	19.0	5.8	15.4	5.7	18.1	6.0	20.6	6.7	16.3	6.8	19.1	7.0	22.7	7.6	19.9	6.9	21.8	7.4
LoE	17.5	4.8	15.6	4.8	17.0	4.9	21.6	5.3	19.3	5.1	20.8	5.3	22.4	5.9	20.9	5.0	21.9	5.6
*Protective factors*																		
SLS	22.5	6.8	25.5	6.8	23.3	6.8	21.5	6.4	22.3	5.9	21.6	6.2	15.9	7.4	19.8	7.9	17.2	7.8
Social Support	64.7	12.6	68.4	14.8	65.6	13.3	62.3	14.0	64.0	17.5	62.8	15.2	51.7	15.8	60.0	13.9	54.4	15.7
Self-control	42.4	9.2	44.0	10.2	44.8	9.4	43.6	7.3	45.0	7.8	42.1	7.4	33.5	8.9	18.0	5.4	34.1	8.5
Resilience	22.1	4.5	22.0	4.7	22.1	4.6	19.7	4.6	16.4	5.9	18.6	5.3	17.3	4.9	18.0	5.4	17.5	5.1
*Offending (N/ %)*																		
Acquisitive	13 (5.2)		2 (2.4)		15 (4.2)		1 (1.8)		0		1 (1.2)		8 (7.7)		0		8 (5.2)	
Drug related	50 (20.0)		6 (7.2)		56 (16.8)		12 (21.1)		2 (6.9)		14 (16.3)		26 (25.0)		9 (18.0)		35 (22.7)	
Violent	20 (8.0)		4 (4.8)		24 (7.2)		6 (10.5)		2 (6.9)		8 (9.3)		19 (18.3)		4 (8.0)		23 (14.9)	
Other antisocial	40 (16.0)		6 (7.2)		46 (13.8)		10 (17.5)		2 (6.9)		12 (14.0)		29 (27.9)		6 (12.0)		35 (22.7)	
Any offence	74 (29.6)		13 (15.7)		84 (25.2)		15 (26.3)		4 (13.8)		19 (22.1)		41 (39.4)		10 (20.0)		51 (33.1)	
PNTS	14 (5.6)		3 (3.6)		17 (5.1)		5 (8.8)		3 (10.3)		8 (9.3)		4 (3.8)		2 (4.0)		6 (3.9)	
Gambling linked^*^	3 (1.2)		1 (1.2)		4 (1.2)		1 (1.8)		0		1 (1.2)		9 (8.7)		0		9 (5.8)	

*DT* dissocial tendencies, *ED* emotional detachment, *DfO* disregard for others, *LoE* lack of sensitivity to emotion, *PNTS* prefer not to say; *participants were asked if any of the offences they have reported committing are directly linked to their gambling involvement

A one-way MANOVA demonstrated a significant difference between those in the behaviourally conditioned, emotionally vulnerable and antisocial-impulsivist groups on the combined dependant variables (i.e. pre-existing anxiety and depression) (*F* (4, 1140) = 138.7, *p *< .001; Pillai’s Trace = .66). When anxiety and depression scales were considered separately, a significant difference between groups was found for anxiety (*F* (2, 570) = 460.6, *p *< .001) and depression (*F* (2, 570) = 132.9, *p *< .001). Tukey HSD post hoc comparison tests revealed that pre-existing anxiety was more severe for those in the antisocial-impulsivist pathway in comparison to the emotionally vulnerable pathway (*p *< .001). There was no difference in the pre-existing depression scores in these pathways.

To test for differences between groups on level of psychopathy, dissocial tendencies, emotional detachment, disregard for others and lack of sensitivity to emotion, a series of one-way ANCOVA’s were performed, with sex as a covariate. There was a significant effect of group on levels of psychopathy (*F* (2, 569) = 53.4, *p *< .001, with a moderate effect size, partial η^2^ = .16). Pairwise comparison, using Bonferroni Adjustment, showed that those within the emotionally vulnerable group reported higher levels of psychopathy than the behaviourally conditioned group (*p* = .02). Furthermore, those in the antisocial-impulsivist group reported higher levels of psychopathy than those in the emotionally vulnerable group (*p *< .001).

There was significant effect of group on *dissocial tendencies* (*F* (2, 564) = 45.5, *p *< .001, with a moderate effect size, partial η^2^ = .14), and *disregard for others* (*F* (2, 565) = 13.1, *p *< .001, with a small effect size, partial η^2^ = .08); those within the antisocial-impulsivist group reported more dissocial tendencies and having less regard for others than those in the emotionally vulnerable and behaviourally conditioned groups.

There was also a significant effect of group on *emotional detachment* (*F* (2, 568) = 20.2, *p *< .001, with a small effect size, partial η^2^ = .06). Antisocial-impulsivist gamblers reported more emotional detachment than behaviourally conditioned gamblers, but not more than the emotionally vulnerable group. Lastly, a significant effect emerged for group on *lack of sensitivity to emotion* (*F* (2, 568) = 58.1, *p *< .001, with a moderate effect size, partial η^2^ = .17). Participants in the antisocial-impulsivist and emotionally vulnerable subgroups reported a greater lack of sensitivity to emotion than those in the behaviourally conditioned group.

Regarding offence type, a series of Chi square analyses examined associations between gambling groups and self-reported offending behaviour. The association between group and acquisitive offending was not significant (*X*^2^ (2, *n* = 573) = 2.4, ns). Further, the association between group and drug-related offending was also not significant (*X*^2^ (2, *n* = 573) = 2.7, ns). The associations between gambling group and violent offending (*X*^2^ (2, *n* = 573) = 7.3, *p* = .027) and other antisocial behaviour offending (*X*^2^ (2, *n* = 573) = 6.5, *p* = .038) were both significant; those in the antisocial-impulsivist group more likely to report having committed violent and other antisocial behaviour offences. However, both had a small effect size (Cramers v = .11).

### Differences Between Groups on Protective Factors

A series of one-way ANCOVA were performed to determine differences between each of the gambling groups on levels of satisfaction with life, social support, self-control, and resilience. Sex was included as a covariate in each ANCOVA.

There was a significant effect of group on satisfaction with life (*F* (2, 569) = 43.36, *p *< .001, with a moderate effect size, partial η^2^ = .13). Pairwise comparison, using Bonferroni Adjustment, showed that those within the behaviourally conditioned and emotionally vulnerable groups reported being more satisfied with life than those within the antisocial-impulsivist group (*p *< .001). A significant effect of group on social support was found (*F* (2, 569) = 35.43, *p *< .001, with a moderate effect size, partial η^2^ = .11). Those within the behaviourally conditioned and emotionally vulnerable groups reported more social support than those within the antisocial-impulsivist group (*p *< .001). A significant effect of group on self-control emerged (*F* (2, 569) = 58.57, *p *< .001, with a moderate effect size, partial η^2^ = .17). Those within the behaviourally conditioned and emotionally vulnerable groups reported more self-control than those within the antisocial-impulsivist group (*p *< .001). There was also a significant effect of group on resilience (*F* (2, 569) = 53.29, *p *< .001, with a moderate effect size, partial η^2^ = .16). Those within the behaviourally conditioned group reported more resilience than those within the emotionally vulnerable and antisocial-impulsivist groups (*p *< .001).

## Discussion

The current series of studies provided evidence that students and those using gambling forums can be placed into three distinct subgroups, based on the presence (or absence) of various sets of psychopathology (Blaszczynski and Nower 2002). These groups can be described as the; (1) *Social Gambler,* whose gambling is driven by social and recreational purposes and whose presentation was characterised by the least severe gambling and an absence of psychopathology; (2) *Affect*-*Regulation* Gambler, characterised by significant affective instability, which is current and also pre-dates their gambling; and (3) *Antisocial Gambler*, characterised by increased levels of impulsivity and antisociality, which manifests in severe multiple maladaptive behaviours.

Evidence for these three groups was consistent with other studies, which have found gamblers can be sub-typed based on typological traits (Blaszczynski and Nower 2002; Nower et al. 2012; Ledgerwood and Petry 2010; Valleur et al. 2016) and include those with an absence of significant pre-existing or current psychopathology (Ledgerwood and Petry 2010; Nower et al. 2012; Stewart et al. 2008; Suomi et al. 2014; Valleur et al. 2016).

The Social Gambler represented the largest group. It was comparable with the behaviourally conditioned group in the Pathways Model (Blaszczynski and Nower 2002). It was supportive, therefore, of the prediction that there would be a cluster of gamblers, similar to this pathway, which were characterised by an absence of notable psychopathology, including offending. It could be speculated that some within the social gambler group could progress across time to become a problem gambler, through processes of conditioning and social learning (Abrams and Kushner 2004; Coventry and Constable 1999). This would be likely determined by (increased) frequency of gambling across time. Increased frequency has been found in longitudinal studies to represent an important predictor of the onset of problem gambling (Williams et al. 2012). It is therefore possible that regular social exposure to gambling could explain why such gamblers’ behaviour could escalate to the point where they could experience problems. However, it could be suggested that for this to occur there would need to be a reinforcing component connected to their motivation for gambling, such as coping and/or a need for stimulation, (e.g. gambling to manage negative emotion, as for the affect-regulation gambler; gambling to enhance stimulation and positive emotions, as for the antisocial gambler). The relative absence of psychopathology in the Social Gambler suggests that a dominating reinforcing component is perhaps absent and/or off-set by increased evidence for protective factors.

The Affect-Regulation Gambler, the smallest group identified, was consistent with the emotionally vulnerable group (Blaszczynski and Nower 2002). This group reported heightened affective instability, both currently and prior to their commencement of gambling, and reported the most negative life events. Consequently, the prediction that there would be a cluster of gamblers consistent with the emotionally vulnerable pathway was supported. Gambling-related cognitive distortions were also more severe in the Affect-Regulation Gambler. There were differences, however, worthy of note between the Affect-Regulation Gamblers within the current research and the emotionally vulnerable group proposed by Blaszczynski and Nower (2002); The Pathways Model suggests that the emotionally vulnerable group includes those who gamble *both* to decrease negative affect and to increase positive affect. However, the current studies suggest that Affect-Regulation Gamblers primarily gamble to cope with negative affect, to *reduce* such affect and escape from their problems. This indicates that those who gamble as a means of *enhancing* positive affect may fall within a different gambler type.

The Antisocial Gambler was consistent with the antisocial-impulsivist group suggested by the Pathways Model. This supported the prediction that such a group would be found and one characterised by offending behaviour and impulsivity. It was further characterised by psychopathic traits. The key difference between Antisocial Gamblers and the other groups appeared that of severity; for example, the Antisocial Gamblers have the same gambling-related cognitive distortions as the other groups, but they are more severe. This gambling motivation also shares important similarities with other studies investigating gambling subgroups. For example, it supports those that have evidenced a group distinguished by increased impulsivity (Ledgerwood and Petry 2006; Suomi et al. 2014; Turner et al. 2008; Vachon and Bagby 2009) and the manifestation of antisocial personality traits (e.g. Ledgerwood and Petry 2010; Nower et al. 2012).

The current research is also one of the first to comprehensively examine antisocial behaviour, with prior research focusing on impulsivity alone (e.g. Lobo et al. 2014; Turner et al. 2008; Vachon and Bagby 2009; Valleur et al. 2016). In doing so, it extends previous literature, and confirms the presence of an antisocial group, by finding that this group also report the most severe dissocial tendencies, emotional detachment, disregard for others and lack of sensitivity to emotion. Thus, the term antisocial-impulsivist, as proposed in the Pathways Model, appears too specific, since the group clearly appears characterised by antisocial tendencies more broadly and not just impulsivity.

Interesting, *enhancing* positive affect also emerged as a primary motivator for gambling in this group. This provided further support that not only does cognition appear as a general thread across all gambling motivations, but so does affect. The latter does not support the Pathways Model, which proposed that antisocial-impulsivist gamblers use gambling as a coping mechanism. Rather, it would appear here that Antisocial Gamblers are simply motivated by a need to enhance affect, which is consistent with expectations of dissocial tendencies, which include a drive for sensation and excitement. It is also consistent with the expectations of psychopathic traits.

It is also worth commenting on the absence of attention to protective factors. The current research has demonstrated a role for protective factors, worthy of recognition. Social Gamblers were found to report the highest levels of protective factors, with Affect-Regulation Gamblers reporting fewer protective factors than Social Gamblers but more than Antisocial Gamblers. Protective factors were clearly identified as a distinguishing feature. The findings were also consistent with the prediction that those aligned with the behaviourally conditioned pathway (i.e. Social Gamblers) would have the highest levels of protective factors, and the Antisocial Gamblers the least. The findings regarding protective factors are unique to the current research. The finding that the Antisocial Gambler had the least protective factors was unsurprising given that this group reported some of the most severe gambling and psychopathology.

**Fig. 1:**
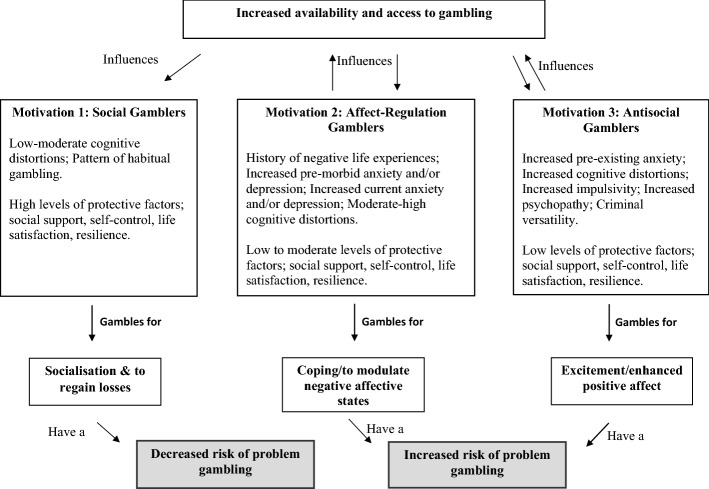
The Integrated Risk and Protective Factors Model of Gambling Types

To account for the findings from the series of studies presented here, an integrated model is proposed. This model, the Integrated Risk and Protective Factors Model of Gambling Types (IRPF-MGT) is unique in its integration of non-problem and problem gamblers into a single applied framework. The principle of multiple factors influencing an escalation of gambling behaviour is apparent in the IRPF-NGT via its inclusion of several important factors drawn from the literature as relevant to gambling. This includes social, cognitive, affect and personality factors. Influenced by the Pathways Model (Blaszczynski and Nower 2002), the IRPF-NGT highlights the importance of exploring the heterogeneity of gambling, whilst also proposing several new contributions. For instance, the refined gambling motivations proposed by the current research (social, affect-regulation, antisocial) begins to address limitations present in the Pathways Model, which focused on pathological gamblers and failed to account for protective factors. In short, the IRPF-MGT proposes three groups of gambler, each associated with specific aetiological processes but containing some common features, distinguishable by testable factors. The IRPF-MGT is presented in Fig. 1.

The IRPF-MGT presents Antisocial and Affect-Regulation gamblers as those most likely to present with increased challenges with gambling, and Social Gamblers with the least level of challenges. This is on the basis of psychopathology absence. There is also a feedback loop indicated in relation to the Antisocial and Affect-Regulation Gamblers, where opportunities to gamble (i.e. increased access and availability) continue to reinforce their engagement. This is not to indicate that the Social Gambler is not similarly influenced, but rather that there is a less noticeable reinforcement component associated with their gambling.

The current research is not, however, without limitations. Participants were invited to recall previous negative life experiences and affective states, prior to their gambling. This retrospective reporting is subject to recall bias and responses may not accurately reflect levels of adversity and premorbid affective states. A longitudinal design would have been able to address these areas more clearly. Self-report measures were also employed to examine the variables of interest but are open to respondent bias; participants may have purposely tried to deceive the researcher, or they may have little insight into or memory of their functioning. The possibility that participants’ responses were guided by a perception, reporting or memory bias must, therefore, be acknowledged. Measures examining socially desirable responding were also not included in any of the studies, but the inclusion of such measures would be of benefit to future research. Finally, there were factors of potential relevance that were clearly not captured. For instance, individuals can have a biological vulnerability for gambling through neurological or neurochemical dysfunction (Boileau et al. 2013; Jazaeri and Bin Habil 2012; Linnet et al. 2011). This research failed to assess for biological vulnerabilities. In doing so, it identifies this as a potential future inclusion for ensuing studies.

Despite the limitations, the current studies suggest a conceptual framework of gambling that is inclusive of risk and protective factors. It is also not attempting to formally diagnose gambling difficulties, instead aiming to provide some insight into the heterogeneity of the concept of gambling. In doing so, the results can offer some suggestions for practice. For example, it is important for clinicians to understand both the spectrum and the heterogeneity of the disorder; awareness of the different types of gamblers and risk factors for gambling could help professionals to understand the full pathology of an individuals’ problems and the need to potentially increase and/or maximise protective factors and/or to disrupt the reinforcing component of gambling. The IRPF-MGT also points to a need to treat other vulnerabilities, in addition to gambling problems, since the latter are potentially a manifestation of other difficulties, such as coping, anxiety, depression and/or dissocial tendencies. Knowledge of such co-occurring issues consequently become important to account for. In addition, due to the varying gambling severity and symptomology among gambling groups, the prognosis and treatment of each will likely differ, indicating more heterogeneity in treatment application. For instance, it is possible that individuals within the Social Gambling group may be able to successfully utilise self-help techniques, should their gambling become problematic. In contrast, those within the Affect-Regulation group may need a more intense intervention that comprises coping strategies and distress tolerance. Those in the Antisocial Group may also require intervention as equally intense as Affect-Regulation gamblers, but this may need to target the core components of dissocial tendencies and raise their protective factors, as for Affect-Regulation gamblers. Future research examining protective factors thus represents a clear direction for research to address. Such research could formally test the Integrated Risk and Protective Factors Model of Gambling Types (IRPF-MGT) proposed here, capturing different problems and gambling severity and attending more to making formal diagnoses of psychopathology, which can avoid some of the challenges of recall biases.
